# Preparation and Functionalization of Polymers with Antibacterial Properties—Review of the Recent Developments

**DOI:** 10.3390/ma16124411

**Published:** 2023-06-15

**Authors:** Monika Parcheta, Magdalena Sobiesiak

**Affiliations:** Department of Polymer Chemistry, Institute of Chemical Sciences, Faculty of Chemistry, Maria Curie-Sklodowska University in Lublin, Maria Curie-Skłodowskiej sq 3., 20 031 Lublin, Poland

**Keywords:** antibacterial polymers, polymer modifications, antibiotic resistant bacteria, wastewater purification, water purification

## Abstract

The presence of antibiotic-resistant bacteria in our environment is a matter of growing concern. Consumption of contaminated drinking water or contaminated fruit or vegetables can provoke ailments and even diseases, mainly in the digestive system. In this work, we present the latest data on the ability to remove bacteria from potable water and wastewater. The article discusses the mechanisms of the antibacterial activity of polymers, consisting of the electrostatic interaction between bacterial cells and the surface of natural and synthetic polymers functionalized with metal cations (polydopamine modified with silver nanoparticles, starch modified with quaternary ammonium or halogenated benzene). The synergistic effect of polymers (N-alkylaminated chitosan, silver doped polyoxometalate, modified poly(aspartic acid)) with antibiotics has also been described, allowing for precise targeting of drugs to infected cells as a preventive measure against the excessive spread of antibiotics, leading to drug resistance among bacteria. Cationic polymers, polymers obtained from essential oils (EOs), or natural polymers modified with organic acids are promising materials in the removal of harmful bacteria. Antimicrobial polymers are successfully used as biocides due to their acceptable toxicity, low production costs, chemical stability, and high adsorption capacity thanks to multi-point attachment to microorganisms. New achievements in the field of polymer surface modification in order to impart antimicrobial properties were summarized.

## 1. Antibiotic Resistant Bacteria in Environment

In recent years, the growing amount of antibiotics in wastewaters constitute a great challenge to the wastewater treatment plants, as the conventional methods—e.g., flocculation, sedimentation, filtration, or coagulation—are not sufficient to remove these pollutants from environment sewage [1]. The most abundant antibiotics found in sewage are trimethoprim, sulfonamides (SA), quinolones, and macrolides, the frequent occurrence of which results from their stability and wide application in the treatment of bacterial diseases in humans and animals [2].

Trimethoprim (TMP) is an antibiotic; its moieties contain electron-rich aromatic rings and a deprotonated amine group, and it is susceptible to the oxidation process, which is proposed as one of the ways to eliminate this compound from aqueous systems [3].

SA are a class of antibiotics that include sulfadiazine, sulfamethazine, and sulfamethoxazole (SMX). One of the possible pathways for the removal of SA is bioaugmentation, which leads to anaerobic degradation of these antibiotics [4].

Quinolones (ofloxacin, ciprofloxacin, norfloxacin) and macrolides (clarithromycin, erythromycin, azithromycin) were recorded in Asia and Europe with occurrence frequencies between 6–30% and 6–10%, respectively [5].

**Figure 1:**
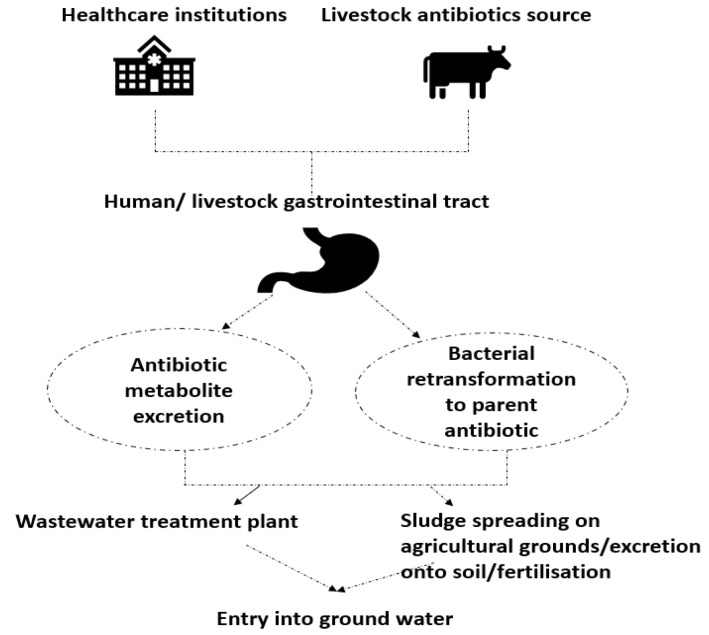
Antibiotic distribution in environment.

The widespread use of antibiotics contributes significantly to the resistance of bacteria to their bactericidal properties, which is a great challenge for modern medicine [6]. An excessive amount of prescribed antibiotics and their limited metabolism in human cells (30–90% of consumed antibiotics are not metabolized in the human body and are excreted into wastewater systems) leads to antibiotic exposure and accelerates resistance in bacteria [7]. The scheme of antibiotic distribution in the natural environment is presented in Figure 1.

Antibiotic resistant bacteria (ARB) acquire resistivity by producing antibiotic resistance genes (ARGs) through the cellular expression process [8]. The capacity of the horizontal transmission of genes in aquatic environments provides an easiness in the spread of antibiotic resistance among humans and animals, which poses a significant risk to health [9]. Antibiotics may enter the environment in the form of metabolites with the retained activity of the original drugs, or they may be excreted from the human/animal body as more polar derivatives of the original antibiotic, which can be then converted by the bacteria into the original drug [10]. ARBs are divided into multidrug-resistant (MDR), extensively-drug resistant (XDR) and pan-drug-resistant (PDR) bacteria, and the criterion for this classification is the number of classes of antibiotics to which the bacteria are resistant. MDRs are bacteria that are resistant to at least one drug belonging to three or more classes of antibiotics at the same time, XDRs are bacteria resistant to at least one antibiotic of each class, except for two or less antimicrobial categories, and finally, PDRs are bacteria resistant to all antibiotics in all antimicrobial categories. MDRs, which have become resistant due to high-volume and long-term use of antibiotics, are of particular concern in Chinese and European intensive care units, where they were responsible for 1.27 million deaths in 2019 [11]. Due to the ability of PDR to effectively withstand all forms of antibiotic therapy, test bacteria pose a particular threat to health facilities. Among the PDR, Gram-negative bacteria can be distinguished, i.e., *E. coli*, *P. aeruginosa*, *K. pneumoniae*, and *A. baumannii*. *P. aeruginosa* is the bacterium responsible for pneumonia, which is particularly common in intensive care units, and poses a particular threat to people suffering from cystic fibrosis and the formation of biofilms. Most nosocomial infections are caused by *K. pneumoniae*, also called the super bacteria, because it has become resistant to all the beta-lactams that make it difficult to treat diseases caused by this bacterium. Some *E. coli* strains may affect the urinary tract, digestive tract, spinal cord, and brain. *A. baumannii* can lead to pneumonia and infections of wounds and the intra-abdomen [12]. Carbapenem-antibiotics-resistant XDR bacteria such as *P. aeruginosa*, *K. pneumoniae*, *and A. baumannii* cause bloodstream infections with high mortality [13].

## 2. Methods of Obtaining Polymers with Antibacterial Properties

**Figure 2:**
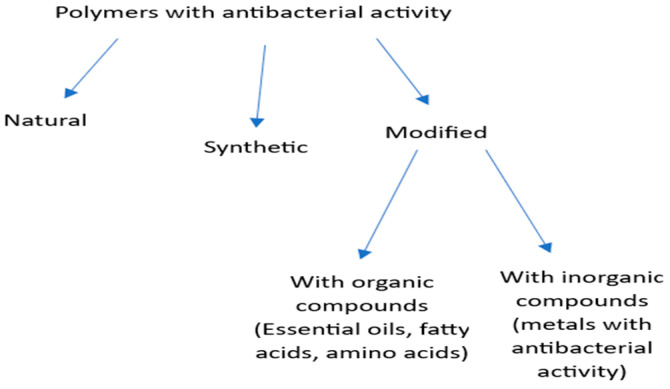
Polymer modification pathways.

Polymers with antimicrobial properties were synthetised for the first time in 1965 by Cornell [14], as the homo- and copolymers of 2-methacryloxytroponone derivatives. Since the 1980s, the host defence polymers and synthetic polymer disinfectants have served as model compounds for the development of the peptide–mimetic antimicrobial polymers of growing recognition. In 1984, the cationic antibacterial polymers based on poly (vinyl benzyl ammonium chloride) were obtained and described for the first time by Ikeda and gained growing interest as effective antibacterial agents [15]. The antibacterial activity of polymers may be their intrinsic feature, but there are also functionalized polymers that receive the biocidal activity through the introduction of active substances such as povidone iodine or N-halamine [16]. Figure 2 schematically shows typical modification routes leading to polymers with antibacterial properties.

### 2.1. Natural Polymers

Among the polymers exhibiting innate antibacterial activity, some natural compounds such as chitosan (CTS) or chitin should be mentioned [17]. Chitosan is a biopolymer consisting of β-(1→4)-2-amino-D-glucose and β-(1→4)-2-acetamido-D-glucose units, known for its outstanding biocompatibility and biological activity; however, its application is limited because it dissolves only in acidic solutions. Chitosan is a product of the deacetylation reaction of chitin, which can be extracted from crustacea, fungi, and insects, and which constitutes one of the most abundant polysaccharides after cellulose. The product of the reaction is considered CTS when the deacetylation degree (DD) of chitin equals about 70%. Further deacetylation, with a DD higher than 95%, may lead to partial depolymerisation. DD influences its biodegradability and physico-chemical properties. The lower the DD value, the higher the molecular weight, which provides better mechanical strength and chemical stability. Unlike most polysaccharides, CTS’s large number of hydrophilic amino groups, which confers CTS a positive charge, renders it likely to form films on the negatively charged surfaces. Moreover, CTS is able to chemically bind negatively charged fats and macromolecules such as proteins. Due to the presence of hydrogen bonds, the structure of CTS is rigid [18,19]. CTS shows high antibacterial activity, but also inhibits the growth of a wide range of fungi. The antibacterial activity of CTS is limited only to acidic environments because of its low solubility at pH levels above 6,5. The biocidal activity of this biopolymer can proceed according to several proposed mechanisms, including inhibiting RNA synthesis by CTS binding to the DNA, and as a result, interfering with the protein synthesis or interaction in the pathogen cell membrane, causing the leakage of proteinaceous material [20]. The other biomacromolecules such as cellulose or starch require chemical modifications to gain any antibacterial activity. Several examples of these modifications are listed in Table 1, which presents the mechanism of antibacterial action of selected modified polymers of natural origin.

### 2.2. Synthetic Polymers

Although synthetic polymers have been known to be inactive and often cause undesired and uncontrolled biological responses when administered to the human body, advances in synthetic techniques have led to new polymers with specific biological applications, including antibacterial activity. One of the examples is a copolymer of maleic anhydride (MA) and divinyl ether (DVE), designated DIVEMA, obtained in a cyclopolymerisation reaction involving an in-chain pyran. DIVEMA shows antibacterial activity against Gram-positive bacteria, Gram-negative bacteria, and fungi [29]. Ilker et al. [30] obtained nonhemolytic, amphiphilic polymers of modified polynorbornes by ring-opening metathesis polymerization (ROMP), under the control of a hydrophilic/hydrophobic ratio. The final product showed antibacterial activity against Gram–positive and Gram-negative bacteria, which can be modulated by the length of the alkyl substituents of polynorbornene [30]. Gabriel et al. obtained copolymers of cationic monomers and of alkyl-substituted polynorborne monomers, with weak antibacterial activity against *S. aureus* and *E. coli* depending on the polymer hydrophobicity, concluding that the balance between hydrophobic and hydrophilic areas is more critical than the overall charge density or global amphiphilicity of polymer to result in biologically active products [31]. Another synthetic polymer with biocidal properties is polyhexamethylene bioguanide (PHMB), which has both a cationic and amphiphilic structure due to the presence of repeated bioguanidine units linked by hexamethylene hydrocarbon chains. Chindera et al. described the antibacterial activity of PHMB as a result of entering bacteria and limiting their chromosomes’ condensation ability [32].

Among the synthetic polymers with antibacterial activity, the poly(dimethylaminomethylstyrene) (PDMAMS) gained recognition as an antimicrobial coating, incorporated on a nylon fabric surface by initiating the chemical vapor deposition (CVD) method. The material was effective against Gram-positive Bacillus subtilis [33]. Tiller et al. [34] described another example of antibacterial polymer coatings applied on a glass surface: N-hexylated poly(4-vinylpyridine) (PVP), which kills more than 99% of *S. epidermidis* and *E. coli*, and more than 90% of deposited *S. aureus*. PVP was obtained through the graft polymerisation of hexyl PVP with 4-vinylpyridine and submitted to the subsequent N-hexylation [34]. The polymers containing nitrogen atoms in heterocyclic rings also display germicidal properties. These pyridinium–type polymers are effective not only against Gram-positive and Gram-negative bacteria, but also against fungi and most yeasts [35]. By analogy, the imidazole derivatives also exhibit antimicrobial activity [36].

The group of synthetic nitrogen-containing polymers with antibacterial properties includes polyethyleneimine (PEI), which is an aliphatic and polycationic compound containing primary, secondary, or tertiary amine groups. Only the branched form of PEI is water miscible, unlike the linear form, which is water insoluble. PEI is effective as an antibacterial polymer against *P. aeruginosa* and *S. aureus* [37]. Highly efficient against a wide range of microorganisms are polyguanidines, which are synthesised in the condensation reaction of guanidine chydrochloride and diamines [38]. The derivatives of guanidine monomers such as polyhexamethylene guanidine (PHMG) and polyhexamethylene biguanidine (PHMB) are applied in interfacial polymerisation reactions with trimesoyl chloride (TMC) on the polysulfone surface, which leads to the formation of ultrafiltration membranes, exhibiting antibacterial activity against *S. aureus* and *E. coli* [39]. Copolymers MMA and PHMG—i.e., poly(MMA-co-PHMG)—are efficient in fighting tap water *E. coli* and heterotrophic-plate-count bacteria [40]. Besides the abovementioned synthetic polymers containing nitrogen atoms, the linear quaternary ammonium polymers (QACs) constitute an important group of antibacterial compounds. The general formula of these polymers is N^+^R_1_R_2_R_3_R_4_.X^−^, where R represents the hydrogen atom or an alkyl group, and X is an anion. The germicidal activity of these polymers is attributed to the long alkyl chain [41]. Due to the low toxicity in relation to mammalian cells and their high stability, QACs find broad applications in the cosmetics and packaging industry. The antibacterial activity of QACs results from the physical interactions of the polymer with bacteria cells, leading to the leakage of the bacterial cytoplasmic constituents such as DNA, RNA, and K^+^ cations. The efficiency of this process depends on many factors, e.g., molecular weight. The higher the molecular weight, the stronger the antibacterial activity. The length of the pendant carbon chain attached to the polycation heteroatom also affects the antibacterial efficiency. The chains with 14–18 carbon atoms showed the highest antibacterial activity against Gram-negative and Gram-positive bacteria [42].

Halogen-containing compounds are gaining increased recognition in the field of antibacterial polymers. Compounds with a halogen atom attached directly to the nitrogen atom are known as N-halamines, which are unique due to their renewable nature, which enables them to be repeatedly charged in the reaction with the halogen donor. N-halamines are synthetised by the halogenation of amine, amide, or imide. In the structure of N-halamines, one or more nitrogen–halogen covalent bonds are present. N-halamines are stable in solutions and effective against a broad spectrum of bacteria [43]. Halogen atoms can be attached not only to the nitrogen atom present in the targeted polymer, but to others as well. The connections between fluorine-containing polymers and quaternary ammonium are also described in the relevant literature and are known for their remarkable antibacterial activity [44]. As in the case of natural polymers, modifications of synthetic polymers can be carried out using organic compounds, i.e., fatty acids or EOs, using metals with antibacterial properties, or by introducing other polymers with intrinsic antibacterial properties into their structure.

### 2.3. Modification of Synthetic and Natural Polymers with Antibacterial Effective Metals

The functionalisation of a polymer surface with metal ions or its oxides is of growing recognition. One of the metals with remarkable antibacterial activity is zinc, whose ions have the ability to inhibit both amino acid metabolism and active transport in bacterial cells [45]. The release of zinc from its oxide nanoparticles (ZnO NPs) is one of the mechanisms of combating prokaryotic and eukaryotic microorganisms. Under light conditions, ZnO NPs exhibit photocatalytic activity and participates in the production of reactive oxygen species (ROS), contributing to the peroxidation of bacterial lipids, damage to nucleic acids, and oxidation of proteins [46]. In addition to antibacterial and bactericidal properties, ZnO nanoparticles are characterized by high catalytic activity and significant chemical and physical stability, which is why they are becoming more and more popular as a polymer matrix modifier. The nanosized ZnO/polymer composites can be obtained via a coprecipitation method described by Matei et al. [47]. ZnO-modified nanostructures obtained by the precipitation method by Rahmah et al. demonstrated excellent antibacterial properties against *Klebsiella* spp. and *S. epidermidis* [48]. Polymer/ZnO hybrids are synthesized by the atom transfer radical polymerization (ATRP) process, which controls the interaction of molecular fillers with the polymer matrix by simultaneously growing all polymer chains at the same rate, which is possible due to the short lifetime of ending radical in any chain [49].

Another important metal with antibacterial properties is silver, used as an antimicrobial agent in all forms or in combination with other technologies. The most popular are silver nanoparticles (AgNPs) with dimensions in the range of 1–100 nm, which have a high surface area to volume ratio compared to silver in bulk. AgNPs show tremendous activity even against multidrug–resistant bacteria, which contributed to its widespread applications in food packaging or the medical industry [50]. AgNPs with dimensions below 30 nm have a better ability to penetrate bacterial cells and show bactericidal activity against *S. aureus* and *K. pneumoniae*. The mechanism of AgNP’s antibacterial action is similar to ZnO nanoparticles and consists of structural changes and deformations of the bacterial cell wall, or in the release and induction of free radicals with a strong bactericidal effect [51]. AgNPs can be obtained and stabilized by physical and chemical methods. The most popular approaches are photochemical reduction, chemical reduction, or electrochemical techniques and irradiation methods. Particularly noteworthy is the polysaccharide method of natural biopolymers modification as an ecological synthesis classified as green chemistry. This synthesis involves polysaccharides acting as a capping agent or acting as both a reducing and capping agent simultaneously. The model example of this modification is the synthesis of starch-AgNPs, where starch was the capping agent and β-D-glucose was the reducing agent [52]. AgNPs are also applied for polymeric membrane modifications. The polysufone membranes can be modified in the wet phase inversion ex situ process of AgNP’s dispersion in the polymer solution [53]. Due to the antibacterial activity of zinc and silver, these metals have particular signification in titanium functionalization. The first application of titanium, discovered in 1790, was limited to an additive in paints; however, its excellent mechanical and chemical properties, such as good corrosion resistance and biocompatibility, contributed to a wider use of this metal in industry and biomedicine, e.g., bone stabilization and fusion and replacement surgery [54]. Apart from silver and zinc nanoparticles, gold and nickel nanoparticles are also gaining interest as antibacterial agent. On the other hand, metal NPs are believed to be toxic to eukaryotic cells in high levels, and their excessive usage may contribute to environmental pollution. Therefore, researchers show an increasing interest in the functionalization of polymers with natural antibacterial agents, e.g., fatty acids and EOs [55,56].

### 2.4. Modification of Synthetic and Natural Polymers with Organic Compounds

**Figure 3:**
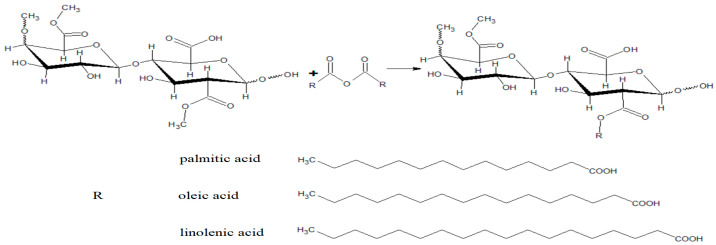
Pectin functionalization with fatty acids anhydrides.

Fatty acids (FA) are organic compounds containing saturated or unsaturated aliphatic chains with a carboxylic group in their linear or branched structures. FAs are produced by algae and plants as an intrinsic protection against pathogens, including multi-drug-resistant bacteria. The antibacterial activity of FA is not well understood yet, but the in vitro tests prove that these compounds are as efficient as Gram-positive and Gram-negative bacteria biocidal agents [57]. Due to the antibacterial activity, fatty acids are often used as synthetic and natural polymer modifiers. The modification of biopolymers with fatty acids can be performed in the reaction of acylation in the presence of K_2_CO_3_ as a catalyst. An example of such a reaction can be the acetylation of pectin with oleic, linolenic and palmitic anhydrides, depicted in Figure 3.

The obtained pectin derivatives are promising antibacterial agents against *E. coli* and *S. aureus* bacteria strains. Moreover, these modified pectin products can be used as coatings in the food packaging industry due to their ability to scavenge oxygen [58]. Decanoic and oleic acids were used for the post-polymerization modification of peptidomimetic polyurethanes based on monomers of N-substituted diols. Prior to the described modification, this synthetic polymer exhibited antibacterial activity against Gram-negative bacteria only. The modification enriched these activities also towards Gram-positive bacteria [59].

Another type of organic compound with intrinsic antibacterial activity are EOs, which are odorous and are a volatile plant’s secondary metabolism product. It has been proven that EOs obtained from different chemotypes such as thyme, mint, cinnamon, sage, and clove have the strongest antibacterial properties [60]. One of the examples of the application of EOs as a modifier is given by Strasakova et al. who obtained antimicrobial polymer film based on a polypropylene modified with EO derived from Carum carvi L seeds via immobilization of the modifier on the carrier of talc in a thermoplastic process [61].

Due to specific features such as amphoteric nature, aqueous solubility, and biocompatibility, the amino acids moieties are also gaining growing attention as modifiers used in this field [62]. According to the place of attachment in the polymer structure, in the 1990s, amino-acid-functionalized polymers were divided in two groups: polymers containing amino acid moieties in the backbone and polymers with the amino acids in the side chains [63]. The incorporation of amino acids in the side chain of polymers enables the formation of higher-ordered self-assembled structures with antifouling properties [64]. The polymers with pendant amine groups can be obtained in various radical polymerization approaches, including atom transfer radical polymerization (ATRP) and reversible addition-fragmentation chain transfer (RAFT) polymerization [65].

**Figure 4:**
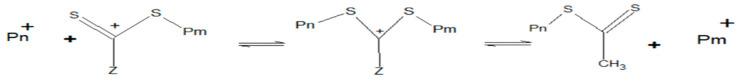
The mechanism of RAFT polymerization.

RAFT polymerization is very common in preparation of the APs due to the high versatility and robustness of this method [66]. The method was elaborated for the first time in the 1990s. Figure 4 presents the scheme of RAFT polymerization. The thiocarbonyls are one of the most often used active agents, which possesses an activating group (Z), modulating the addition and fragmentation rates and a radical leaving group (R), which must be able to efficiently reinitiate the polymerization process [67].

RAFT-derived polymers are functionalized with reactive-end groups, which enables coupling of bioactive macromolecules. Products of RAFT polymerization have a controlled molecular weight and low polydispersity index. Asif et al. [68] synthetized novel diblock copolymers of vinyl acetate with comonomers such as 4-vinylbenzyl chloride, 1,3-divinyltetramethyldisiloxane, or vinyltriphenyl phosphonium bromide using cyanomethylmethyl(phenyl) carbamodithioate as RAFT agents. The copolymers showed antibacterial activity against *S. typhi*, *P. aeruginosa*, *B. subtilus*, and *S. aureus* bacterial strains [68]. By using RAFT, Yadav et al. obtained the antibacterial zwitterionic poly(cysteine methacrylate), which also exhibited antibacterial properties against Gram-positive (*R. erythropolis*) and Gram-negative (*E. coli*) bacteria [69].

**Figure 5:**
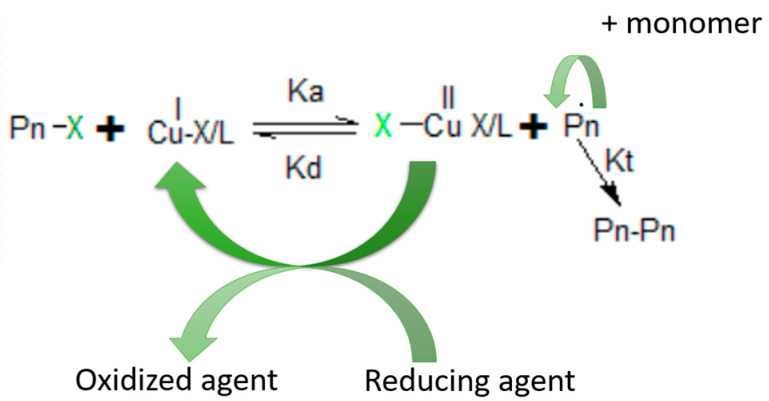
Mechanism of ATRP polymerization.

ATRP is also a method of controlled radical polymerization, but in comparison to RAFT polymerization, ATRP requires a transition metal catalyst, which is harmful to biological systems. The ATRP reaction has to be conducted in the inert atmosphere, which constitutes one of the major disadvantages of this process [70]. In Figure 5, the mechanism of ATRP polymerisation is presented.

The scheme above presents the reaction between the initiating alkyl halide and transition metal complexes in a lower oxidation state, coordinated with ligand L. The products are growing radicals P_n_* and metal complexes with a higher oxidation state [71]. The ATRP method was applied to obtain the antibacterial cellulose derivative, which had acceptable antibacterial activity against *Escherichia coli*, *Salmonella enterica*, *Staphylococcus aureus*, and *Bacillus subtilis* [72].

Furthermore, click chemistry attracted the attention of scientists searching for antibacterial macromolecules. Click chemistry was introduced for the first time by Sharples et al. and Meldal in 2001 as a method for linking some units together using a heteroatom C-X-C [73].

Currently, these six types of click reactions are proposed for post polymerization modifications:

− Michael addition,
− Diels–Alder reaction,
− imine formation,
− alkyne-azide reaction,
− epoxy–amine/thiol reaction and
− thiol–ene reaction [74].

The two-step thiol–ene click reaction was applied to obtain a poly[(mercaptopropyl)methylsiloxane] (PMMS)-based polymer with 100% killing proficiency against *Staphylococcus aureus* [75]. In turn, a copper (I)-catalyzed azide-alkyne cycloaddition “click” reaction (CuAAC) was conducted by Acik et al. in order to obtain an antibacterial film from chlorinated polypropylene, with activity against *E. coli* and *S. aureus* [76]. The selected examples of applications of modified polymers are presented in Section 3, Section 4, Section 5 and Section 6.

## 3. Polymeric Materials with Antibacterial Properties—Mechanism of Action and Medical Applications

**Figure 6:**
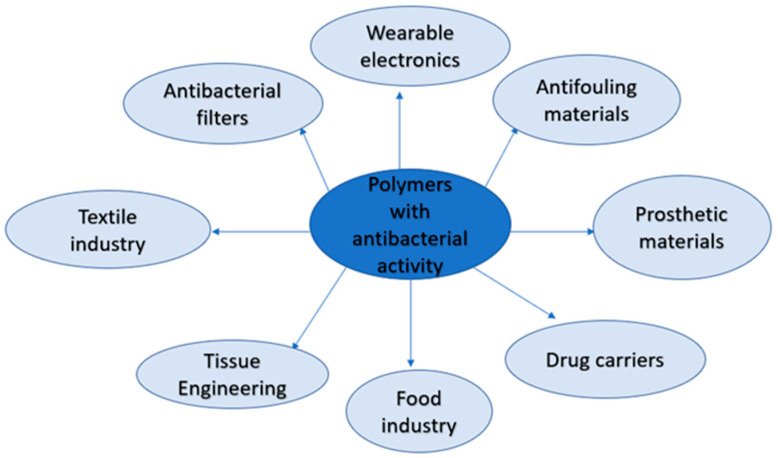
The industrial application of polymers with antibacterial activity.

It is predicted that antibiotic-resistant bacteria can result in 10 million deaths by 2050 [77]. The issue of multidrug-resistant bacteria urged the need to elaborate the novel antipathogen agents [78]. The polymers and copolymers modified with bioactive compounds have emerged as a group of highly effective antimicrobial agents [79] that find usage in many fields (Figure 6).

The factors that are of the greatest influence on the antimicrobial properties of bioactive polymers are their low toxicity towards human cells and high activity in fighting bacteria cells [80]. The mechanism of antibacterial activity of these compounds is provided by active and passive ways of interacting with pathogens [81]. The active mechanism of disrupting the function of bacteria cells consists of the destabilisation of bacteria cells through electrostatic interactions between the predominantly hydrophobic and negatively charged bacteria plasma membrane, and the positively charged surface of the modified cationic polymer [82]. The most popular active substances used to modify the surface of polymers are quaternary ammonium salts, which interact with the negatively charged membranes of bacteria, causing leakage of components out of the bacterial cell, and consequently, the cell’s death [83]. Similar mechanisms of active functional disturbance by electrostatic interaction are exhibited by polyethylenimines. Another example of modifiers on the polymer surface are polyguanidines, which inhibits the bacterial growth due to it breaking the Ca^2+^ salt bridges and N-halamine, which disrupts the function of the amino cell receptors in bacteria by generating the oxidative halogen [84]. The passive mechanism of fighting bacteria cells relys on the synthesis of the passive polymer layer, which prevents the adhesion of bacteria on the modified polymer surface, thereby repelling the bacteria without any active interaction with them [85].

The natural polymers have a great advantage over the synthetic ones due to their non-toxicity, biocompatibility, non-immunogenicity, and high stability. On the other hand, they are less effective in biomedical applications in comparison to synthetic polymers [86]. The modifications that provide the natural polymers with desirable industrial activity include chemical treatment processes such as hydroxylation, carboxylation and epoxidation, or in vitro enzyme treatment [87].

Synthetic polymers frequently used in the synthesis of polymers with antimicrobial activity are based on poly(lactic acid) (PLA), polyethylene glycol (PEG), and polyamides [88].

### 3.1. The Medical Application of Polymeric Materials with Antibacterial Activity

The polymers with antibacterial activity are applied in medicine as drug carriers. The encapsulation of the drugs into micelles, nanogels, or vesicles [89] not only allows it to curb the bacterial resistance to antibiotics, but also increases the bioavailability of the drug compared to the same conventional antibiotic.

For medical purposes, natural (alginate), artificial (CTS, ethyl cellulose (ET)), and synthetic (PCL—poly(epsilon-caprolactone), PDLA—poly(D-lactide), PGA—poly(glycolide), PLA, PLGA—poly(lactic-co-glycolic acid) polymers are used. The choice of polymer applied as a drug carrier is determined by required residence time and administration site in human cells [90]. In addition, the toxicity and tolerance of the polymer carriers in the relevant cell type is assessed.

The polymers applied in drug delivery systems should have hydrolytically or enzymatically cleavable chemical bonds that provide biodegradability in the body, although the non-biodegradable polymers such as polymethacrylates also constitute a promising alternative [91,92,93].

One of the most innovative drug delivery approaches involve the polymer nanoparticles (NPs) [94]. The NPs, due to their nanometric dimensions (1–100 nm), are easily accessible to cells and tissues, and deliver a drug straight to the site of action in the human body [95]. The NPs are synthetised in the form of nanospheres or nanocapsules. The main difference between them relies on the placement of the carried drug and the mechanism of drug incorporation. The nanospheres are colloidal particles, which adsorb the drug molecule on the particle surface, while the nanocapsules take the form of surrounded polymer shell vesicles with the core filled with aqueous or oily liquid in which the drug is dissolved [96]. Among the nanocapsules, dendrimers, micelles, liposomes, and polymersomes are used as nanoparticles to deliver drugs, including antimicrobials [97]. Lipid constructs called liposomes are composed of bilayers made of amphipathic lipids. Natural liposomes can be found and isolated from the cells, but synthetic liposomes also are commercially available. Due to the presence of the aqueous phase inside and between the lipid bilayers, they can deliver both lipophilic and hydrophilic drugs to human cells [98].

Dendrimers, synthetised for the first time in 1978 by Vögtle et al., are the smallest among the NPs, with a diameter between 1–10 nm. They are obtained in the reaction of protection–deprotection synthesis of the hyperbranched macromolecules, followed by the elongation of the bioactive site from the multifunctional core molecules [99]. Dendrimers are particularly interesting as drug carriers due to their amphiphilic structure, globular shape, low dispersity, and highly branched three-dimensional structure [100]. The bioactive sites of dendrimers are formed by their surface functional groups, and can be modified with biologically active antimicrobial groups, which provide antimicrobial activity to the polymer [101]. The interaction between the bacterial cell and the modified dendrimer surface take place through the electrostatic interactions. Negatively charged bacterial cells interact with positively charged dendrimer functional groups, increasing the permeability of the cell membrane and contributing to the biocidal effect [102]. PAMAM—poly(amido)amine, dendritic polylysine, and polypropylenimine (PPI) [103] are the most popular, commercially available dendrimers with a cationic surface.

Polymersomes are amphiphilic bilayer vesicles made of tri- or di-copolymer blocks, whose properties are crucial for the overall features of the obtained vesicle. In comparison to liposomes, the polymersomes exhibit greater structural and mechanical stability [104]. However, the mechanism of drug transportation is similar—the water-soluble molecules are carried in the inner space of vesicle while the hydrophobic molecules are transferred in the bilayer [105].

**Figure 7:**
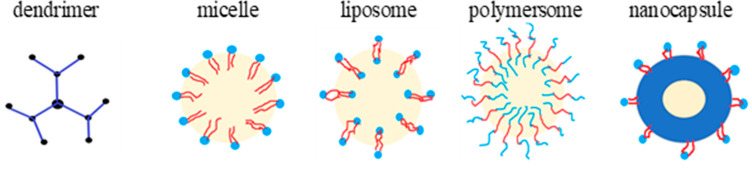
The schemes of polymer nanoparticles. Blue colour represents the hydrophilic part of the NPs; red colour represents the hydrophobic one. The space in which the drugs are introduced is marked in yellow.

Polymer micelles are vesicles with a lipophilic core in which only the hydrophobic drugs can be encapsulated, and a hydrophilic shell ensures water solubility of the entire particle. On the contrary to polymersomes, micelles are not able to transport the hydrophilic drugs [106]. Figure 7 shows a schematic representation of various drug nanocarriers.

Polymer nanoparticles in combination with antibiotics can also be used as synergistic or additive agents to chemically or physically weaken the bacteria via the use of elevated temperatures or the formation of reactive oxygen species. Gold NPs, thanks to their high photothermal efficiency in the presence of near-infrared radiation, are of particular clinical interest because exposure of bacteria to temperatures in the range of 45–50 °C causes a strong antibacterial effect in the body in the form of an increase in the level of cytokines and the body’s cellular immune response. The photothermal NPs may be incorporated in the structures of microneedle (MN) arrays, enhancing the antibiotic delivery directly to the site of infection. MNs are obtained from soluble polymers, making them suitable for delivering antibiotics in a humid environment, and providing high local concentrations of antibiotics to infected cells of the human body. Among the antibiotics successfully delivered by the MNs are vancomycin, polymyxin, tetracycline, chloramphenicol, clindamycin, cephalexin, doxycycline, and gentamicin. Further examples of NPs exhibiting a synergistic effect with antibiotics are tetracycline, chloramphenicol, and rifampicin, which are N-alkylaminated CTS NPs that showed high efficiency against Gram-negative bacteria (*E. coli*, *S. typhimurium*). In comparison to metal NPs, these natural mucopolysaccharide NPs are considered more biocompatible and biodegradable, but at a concentration higher than 200 mg/L, chitin nanoparticles exhibit cytotoxic properties [107]. Photothermal antibacterial treatment gained recognition due to the reduction of side effects in tissues, low toxicity, high selectivity, and the lack of drug resistance [108]. One of the main disadvantages of photothermal treatment of bacterial infections is the necessity for application at a high temperature to make this treatment efficient against drug-resistant bacteria. In order to eliminate this issue, the photothermal treatment can be replaced with chemodynamic therapy with a synergistic effect. The environment of the infection site is characterized by a low pH and overexpression of H_2_O_2_, which allows for precise targeting of the drug. The application of the silver-doped polyoxometalate (AgPOM) injectable in situ hydrogels are one of the most direct infection-targeting methods, featuring good tissue adhesion, a long-lasting effect, good repeatability, and great photothermal performance [109]. Wounds infected with bacteria can also be successfully treated by using hydrogels prepared from poly(aspartic acid) modified with a quaternary ammonium compound/boronic acid cross-linked with poly(vinyl alcohol) polymers. These hydrogels reduced epidermal bacterial survival to 2.3% with an optimal healing rate of 92% after 7 days [110].

### 3.2. Polymer Materials as Antifouling Agents

Apart from drug delivery and antibacterial treatments, the natural and synthetic nanocomposites are applied in cancer therapy, dental applications, and tissues engineering [111].

**Figure 8:**
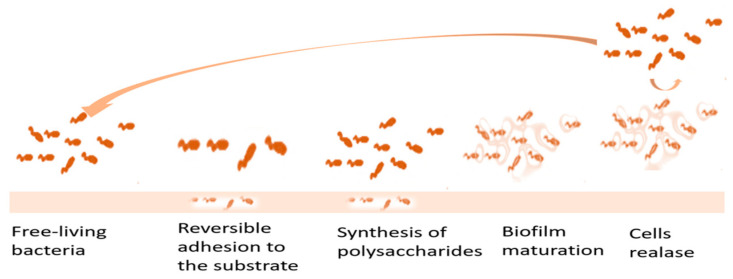
The scheme of biofilm formation. Arrow describes the circulation of bacteria cells.

Bacterial infections, apart from mechanical damage, are one of the main causes of transplant failures. Polymeric biomaterials are often used as an antibacterial surface in regenerative medicine, and as the coating for medical implants that prevent bacterial biofilm generation. Bacterial biofilm formation is a defence mechanism against host immune cells, ensures chronicity of infection, and is initiated by a bacterial recognition known as quorum sensing. According to statistics, as much as 80% of clinical infections in humans are caused by biofilms. The presence of biofilm is often observed on the surface of orthopedic screws made of stainless steel and titanium. A mechanism of biofilm formation is presented in Figure 8. The first step is the bacteria;s adhesion to the surface. At a distance of about 50 nm from the implant surface, bacterial cells are attracted by Van der Waals forces. At a distance of 20 nm, between the bacteria and the implant, electrostatic repulsive forces occur depending on the interaction between the surface and the usually negatively charged bacteria. At 5 nm from the surface, the strongest Van der Waals and electrostatic forces, as well as hydrophobic and site-specific interactions, begin to occur. After adhesion to the surface, bacteria start to proliferate and grow, producing extracellular polymeric substances that help them capture nutrients and improve their survivability. Due to the cell–to–cell communication in biofilm, bacteria are able to adapt to environmental conditions and colonize new surfaces. After biofilm maturation, some of it dissipates, releasing floating bacteria that can redeposit on the surface [112,113,114].

**Figure 9:**
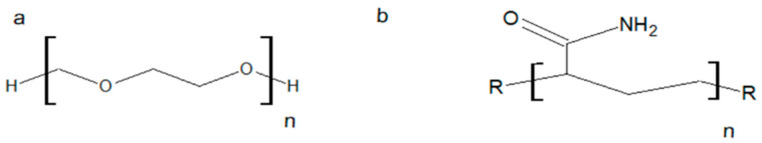
The condensed formulas of PEG (**a**) and PAM (**b**).

Among the polymeric materials that can be helpful in preventing biofilm formation are antifouling polymers, which repel the bacteria from the surface with chemical or physical mechanisms, and antibacterial ones, e.g., peptide mimetic polymers and cationic polymers [115]. Within the antifouling agents, the surfaces functionalised with hydrophilic, zwitterionic, and superhydrophobic polymers should be listed [116]. The feature of hydrophilic polymers is their favourable interaction with water, which provides them good solubility and swellability [117]. In transplantology, hydrophilic polymers are of great interest due to their ability to mimic the properties of natural cartilage [118]. Poly (ethylene glycol) (PEG) and poly (acrylamide) (PAM) (Figure 9) are popular representatives of such hydrophilic polymers [119].

An alternative to hydrophilic polymers such as PEG is zwitterionic polymers, which shows better antifouling properties [120]. Zwitterionic polymers also exhibit significant chemical and thermal stability, and excellent biocompatibility even in complex surroundings, e.g., serum or blood [121]. The structure of these polymers mimics natural compounds occurring in human cells, such as glycine betaine [122]. The repeating constitutional units of zwitterionic polymers contain both negative and positive charges which make them electrically neutral and hydrophilic; furthermore, the entire network of such a polymer exhibits the same characteristics (electrical neutrality and hydrophilicity) [123]. On the other hand, the hydrophilicity of zwitterionic polymers is one of their greatest disadvantages, as it leads to a strong absorption of water. High solubility in water and susceptibility to hydrolysis limit their ability to form a film, and as a result, it curbs the use of these polymers as antifouling agents [124]. To overcome this difficulty, cross-linking molecules such as polydimethylsiloxane (PDMS) are used to form thin zwitterionic films [125].

The most popular zwitterionic polymers are polybetaines. The positive charge in their monomeric units is provided by a quaternary ammonium group, while the negative one is related to the presence of the anionic groups such as sulfonates, carboxylates, phosphonates, phosphates, and phosphinates. According to the charge distribution mode, apart from polybetaines, among the zwitterionic materials, the polyampholytes are also distinguished. The main difference between polybetaines and polyampholytes is the position of the charge. Polybetaines have both cationic and anionic groups located on the same monomer unit separated by an alkyl chain, while polyampholytes have their negative and positive charges situated on different monomer units [126].

Another type of bacteria-repelling molecule is superhydrophobic polymers, inspired by lotus leaves, covered by hydrophobic wax. The fluorinated silica-colloid-based surfaces are an example of superhydrophobic polymers exhibiting antiadhesive activity towards *S. aureus* and *P. aeruginosa* [127]. There is also growing interest around titanium-based materials. Their superhydrophobic properties, bioavailability, and favourable mechanical properties make them useful for cardiac implants [128]. In orthopaedics and dentistry, the magnesium alloy coated by hydroxyapatite (HA) and stearic acid confer great antibacterial adhesion capacity [129]. The polymers applied in regenerative medicine and tissue engineering must cope with the changes of the extracellular environment that accompany physiological and pathological processes.

Chemically synthesized materials that mimic the extracellular matrix (ECM) appear to be a promising approach to imitate the biological activity of cells [130]. The ECM are mostly composed of proteins that perform essential functions in biological processes such as enzymatic reactions, immunological response, cells motility, or signal transduction [131]. Thus, protein-mimetic polymers offer hope for accessing complex natural mechanisms. The amphiphilic polymers imitating antimicrobial peptides (AMPs) are highly efficient in preventing biofilm formation. One example of AMPs is photoresponsive AMP based on the N-substituted glycine skeleton, which—due to its efficiency, controllability, and high selectivity—has been used in hydrogels and antifouling surfaces [132].

Another kind of polymeric material used as an antifouling agent are the cationic polymers mentioned above, which have been proved to exhibit excellent antibacterial properties. In implantology, polyurethane catheters are often used as implantable medical instruments. Unfortunately, their surface is susceptible to the adhesion of bacteria, which necessitates their frequent replacement in order to prevent bacterial infection. To thwart the formation of a biofilm on polyurethane catheters, the surface modification with cationic polymers can be applied. For this purpose, quaternary ammonium compounds or metal ions are used [133].

Some examples of a quaternized compound are benzophenone-based esters and benzophenone quaternary amides, which can be cross-linked on surfaces upon UV radiation. These coatings are efficient against the methicillin-resistant *Staphylococcus aureus* (MRSA), fluconazole-resistant *Candida albicans* spp., and influenza virus with 100% efficiency [134]. A modified (quaternized or alkylated) polyethyleneimine (PEI) is a cationic polymer containing amino- and imino-groups, also known for its antibacterial properties. PEI is positively charged in neutral and basic solutions, having a high zero potential point at pH values up to 10 [135].

The cationic polymers can also be obtained in the innovative reaction of photopolymerization. This method is applied to prepare Sulphur-containing polymers, whose monomers are ionized giving a positive charge, which provides the polymer with antifouling properties [136].

## 4. Antibacterial Water Filters

One of the biggest problems facing developing countries is the treatment of tap water, as microbial contamination makes it unfit for drinking [137]. For the sake of human health, water treatment devices with antibacterial and adsorptive properties are of great importance and are the subject of our further research. The aim of our future research will be the development of non-polluting, highly efficient, and environmentally friendly antibacterial filters modified with natural organic acids.

Drinking contaminated water for a long time can contribute to a decrease in immunity or even malnutrition. One of the most frequently chosen methods of preventing microbial contamination are membrane filters. Among the most commonly used filtration materials are zeolites, activated carbons, and resins. Hu et al. prepared an antibacterial composite based on CTS/biocarbon nanosilver (C-Ag), obtained by carbonization of corn straw as a carbon substrate, with good adsorption capacity towards metals such as Cu, Cd, Zn, and Pb, and antibacterial activity against *B. subtilis* and *E. coli* [138].

The mechanism of operating membrane filters is often based on popular purification processes: reverse osmosis (RO), forward osmosis (FO), nanofiltration (NF), microfiltration (MF), and ultrafiltration (UF), which differ in operating procedures and pore sizes. High thermal and chemical resistance as well as low manufacturing costs contribute to the growing interest in polymer membranes, such as polysulfone, polyether sulfone, polypropylene, and polyvinylidene fluoride. However, biofouling on these materials significantly reduces their direct use in the water treatment process.

Since conventional methods such as physical or chemical cleaning of membranes were developed, the pre-treatment of feed and dosing of biocides are not effective in preventing biofouling. New membranes with antibacterial properties are obtained by modification of the surface to control membrane biofouling. The modifiers can be antibacterial agents such as N-halamine, pyridine, and bio-enzymes [139].

N-halamines are organic or inorganic compounds, containing at least one covalent bond between nitrogen and halogen. The mechanism of antibacterial action is related to the hydrolysis of the nitrogen-halogen bond and its reduction to the nitrogen-hydrogen group. Oxidative halogens released in this reaction directly affect the viability and metabolism of bacteria [140]. Shao et al. [141] used N-halamine to modify a polyvinylidene fluoride (PVDF) nanofiber membrane, which showed persistent antibacterial properties, providing a new idea for water filtration. The PVDF exhibited antibacterial activity against *S. aureus* and *E. coli* [141]. N-halamine has also been applied for the decoration of electrospun porous polyacrylonitrile nanofibrous (PAN) membranes, which had promising antimicrobial activity against *K. pneumoniae* and *E. coli*. These membranes were obtained via electrospinning and soaking methods. In the electrospinning technique, N-halamine is mixed with the polymeric solution of PAN, while the soaking method relies on immersing the electrospun PAN nanofibers in an N-halamine solution [142]. For water treatment, polymers with pyridine quaternary ammonium salts moieties are also used in the form of hollow nanocapsules, which kill bacteria using a similar mechanism as polycations [143].

In order to control biofilm formation, membranes for water treatment can be also modified with bio-enzymes, whose role is to enzymatically clean the membrane surface. The main advantages of this cleaning strategy are the selectivity, specificity, and non-toxicity of the enzymatic degradation products [144].

Another way to prevent the adhesion of planktonic bacteria and formation of a biofilm is to use a sulfonated pentablock copolymer (s-PBC) as a coating for water filters. s-PBC can be used as a coating for polypropylene (PP), providing a more hydrophilic and negatively charged surface. The resulting water filters are particularly effective against *P. aeruginosa*, which is responsible for most nosocomial infections, since the bacterial cells form a biofilm on the taps, contaminating outflowing water and increasing the risk of infection [145].

In the case of industrial-scale water treatment, modification of membranes with nanocomposites is gaining importance. The obtained products are known as mixed matrix membranes (MMM), or nanoparticle-reinforced membranes.

The nanocomposite membranes are not only highly selective, self-cleaning, and easy to use, but also show a high resistance to contamination. Silver-based nanoparticles are one of the most common modifiers, but nanoparticles of titanium (TiO_2_), copper (CuO), graphene (GO), iron (Fe_3_O_4_), silicon (SiO_2_), aluminium (Al_2_O_3_), zinc (ZnO), and zirconium oxides (ZrO_2_) are also popular. The polysulfone (PSF) membranes, coated with TiO2-NPs, GO, and ZnO, are known for their excellent antifouling properties for water treatment and for their antibacterial properties [146]. ZnO and ZrO_2_ incorporated to polymeric nano-composite materials show enhanced hydrophilicity, porosity, high permeability, and antifouling properties. ZnO also has great ability for metal ion adsorption, e.g., Cu^2+^. The incorporation of ZrO_2_ into a polysulfone membrane with tin dioxide (SnO_2_) by the sol-gel method also improved antifouling behaviour. Graphene oxide (GO) is obtained by the oxidation of graphene, which leads to the presence of oxygen-containing groups, giving GO an amphiphilic character. The exact mechanism of the antibacterial action of GO is not known yet, but it is assumed that bacterial growth is inhibited by ROS (Reactive Oxygen Species) generation. Although graphene oxide itself is considered to have antibacterial activity, examples of the metal modification of GO have also been described [147]. GO modified with quaternary ammonium salt (QGO) has been used to modify PVDF, which contributed to a significant reduction in the loss of antibacterial substances from the membranes. Carboxyl and epoxy groups can be involved in the grafting of quaternary ammonium salts. PVDF membranes modified with QGO are obtained using the liquid-solid phase transformation method [148].

Other quaternary ammonium salts, e.g., diallyldimethyl ammonium chloride (DADMAC), were also applied for modification of the polyamide composite, which revealed long-term biofouling resistance. The biocidal activity of the obtained DADMAC-modified membrane is a result of the interaction of hydrophobic alkyl with the hydrophilic group in bacteria cells, changing their permeability and resulting in cytolysis [149].

For the purposes of ultra-purification of water, silver nanoparticles gained great interest due to their low toxicity and microbial resistivity. Ag-NPs can be embedded in hollow membranes, rendering them with biocidal properties. The low production costs and high mechanical stability made the hollow membranes a promising solution for ultra- and micro-filtration [150]. Incorporation of Ag-NPs in a cellulose acetate (CA) hollow membrane leads to the improvement of biocidal effects against *E. coli* and *S. aureus*. The nanoparticles of CuO exhibit biocidal activity towards methicillin-resistant *S. aureus* and *E. coli*, with minimum bactericidal concentrations. A mechanism of the antibacterial action of CuO NPs is also not well described in the literature yet. One of the possible mechanisms assumes the releasing of copper ions from nanoparticles, which causes damage to the bacterial cell membrane and even incorporation into bacterial DNA. This interaction contributes to the disruption of the helical structure of DNA. The last probable mechanism is related to the generation of ROS, which is responsible for giving rise to oxidative stress and ultimately damaging bacterial cells [151].

Membrane-based technologies for water treatment also take advantage of modification with organic compounds, e.g., tannic acid (TA) and polyhexamethylene guanidine (PHMG). Both of these compounds are environmentally-friendly antimicrobial modifiers, applied as a coating for PVDF porous membrane [152].

## 5. Antibacterial Polymers in Food Packaging

According to the newest data published by the WHO in 2010, foodborne illnesses contribute to 420,000 deaths globally [153]. The main cause of disease are pathogens that multiply on the surface of food and its packaging. To prevent food from contamination, innovative packaging that forms a barrier between the food and the external environment is essential. The stringent requirements that must be met by packaging materials used to ensure a longer shelf life of food include antibacterial activity, non-toxicity, and impermeability [154]. Among the materials used to produce food packages, fossil–based polymers such as polyvinylchloride (PVC), PET (poly(ethylene tetraphtalate)), polyethylene (PE), and PP are the most common, but the tendency to replace them with biodegradable biopolymers, such as CTS, starch, or cellulose, is growing.

PVC is one of the most popular thermoplastics, utilized in production of antibacterial packaging due to its great softness, flexibility, chemical inertness, and excellent self–cleaning properties in processing. The great easiness in processing is also exhibited by PET, which is one of the most common substitutes of PVC in food packaging applications. PET is well known for its good mechanical strength and toughness. In turn, PP is a thermoplastic material obtained in the process of propylene polymerization. Due to its high melting point, it can be applied in packaging processes conducted under high temperatures [155].

Natural polymers such as CTS gained attention in the food industry as packaging films due to their mechanical and barrier properties, which prevent food spoilage by limiting the permeability of oxygen, carbon dioxide, water, and organic vapour or liquids [156].

Another biopolymer used in the food packaging industry is starch, due to its high biodegradability, low cost, and natural abundance. The botanical sources of this biopolymer are potatoes, maize, or water chestnut. The molecular structure of starch is complex and consists of amylose and amylopectin [157]. The higher the percentage of amylose in regard to amylopectin, the higher the tensile strength (TS) and elastic modulus (EM) parameters, which describe the robustness of starch. In contrast to other polysaccharides, starch has the ability to become a thermoplastic when food-safe plasticizers are added, including glycerol, xylitol, and sorbitol [158].

An important biopolymer that can be inexpensively acquired from agricultural waste and forest residues is cellulose. Cellulose is a natural component of the cell walls of plants. Chemically, it is a high-molecular-weight homopolymer of β–d-glucopyranose units linked by β-1,4 linkages, where the cellobiose is the repeated unit. Due to its high thermal stability, cellulose can be used as a shield against ultraviolet rays. Cellulose esters and ethers are also of great importance in the packaging and food industry, e.g., cellulose sulphate (CS), CA, EC, and methyl cellulose (MC) [159].

There are four different strategies for incorporating antimicrobial agents into food packaging material, e.g., incorporation and coating onto polymeric film, and also releasing antimicrobial agents from the sachets and direct contact from pads [160]. The antibacterial agents used to modify polymeric materials to obtain food packaging include compounds such as proteins and peptides, polyphenols, EOs, organic acids, and natural extracts [161].

In accordance with European regulations for materials intended to come into contact with food, intelligent packaging must be compliant with Regulation 1935/2004/EC, which says that packaging material should meet the requirements regarding overall migration limits (OMLs) and specific migration limits (SMLs) in order to prevent the migration of packaging ingredients towards food [162].

Phenolic acids are used as antimicrobial agents incorporated into the packaging film. Compared to EOs, phenolic acids are non-volatile and have a lower organoleptic effect on food matrices. They exhibit antimicrobial activity against Gram-positive and Gram-negative bacteria, inhibiting cell growth due to their passage in the protonated form to the plasma membrane. After encountering the higher pH within the bacterial cell, cations and anions liberated in acid dissociation are unable to pass through the cell membrane [163]. Among the phenolic acids used to modify packaging, ferulic acid and cinnamic acid should be mentioned. They can be incorporated into packaging films by solution spraying. Due to the high solubility of these acids in ethanol, it is easy to obtain crystal structures that can be incorporated into the polylactide surface [164].

Ferulic and cinnamic acids are also applied to obtain plasticized cassava starch films by melt blending and compression moulding. The addition of these acids to starch leads to the formation of more extensible, less water-soluble, and breakage-resistant membranes that inhibit growth of *E. coli* and *L. innocua* [165]. The incorporation of ferulic acid into pullulan-based composite films using a solution-casting method also leads to the antioxidant and biodegradable bioactive packaging [166].

Active packaging with the controlled release of ferulic acid was also made of low-density polyethylene (LDPE) and ethylene-vinyl acetate (EVA) [167]. Apart from the acids described above, sorbic, syringic, boswellic, citric, or tannic acids are also used as modifiers. Since 1940, sorbic acid has been known for its excellent antibacterial activity. In addition, this compound swiftly decomposes in the soil, which makes it environmentally friendly. Sorbic acid is also employed commercially as a preservative agent, designated as E200. Active polypropylene films containing sorbic acid show antibacterial properties against *E. coli*, *S. aureus*, and an antifungal effect on *A. niger* [168].

CTS films, with the addition of citric acid as a cross-linking agent and glycerol as a plasticizer, have great packaging stability. Additionally, the final products show good antioxidant properties [169]. CTS-based active packaging can also be obtained by including boswellic acid (BA), which is a pentacyclic triterpene acid obtained from the resin of deciduous trees from the dry regions of India and China. BA is known for its anti-inflammatory and anti-cancer properties. Packaging films produced out of CTS, PVA, and BA (CPBA films) show activity against *E. coli*, *S. aureus*, and *Candida albicans* [170].

Composite films based on CTS and syringic acid also have a significant bacteriostatic effect against *E. coli* and *S. aureus* bacteria. Syringic acid belongs to the family of phenolic acids, which have great antioxidant properties and can be found in *RadixIsatidis* and *Lenntinulaedodes* [171].

Methylcellulose, CTS, and gelatine films doped with tannic acid, which is a gallic ester of D-glucose, also exhibit antibacterial activity against the bacteria mentioned above. Apart from antibacterial activity, tannic acid also exhibits antioxidant properties due to the presence of phenolic groups. As a compound generally recognised as safe (GRAS) by the Food and Drug Administration (FDA), tannic acid is commercially used in the food industry [172]. GRAS compounds that can be successfully applied to development of antimicrobial food packaging materials also include natural extracts and phytochemicals, e.g., *Murta fruit*, green tea, spirulina, citrus and propolis extracts, which all have antimicrobial activity.

Antimicrobial packaging materials can also contain EOs. Their bacteriostatic properties result from their hydrophobicity, which increases the permeability of cells and mitochondrial membranes, contributing to the leakage of small molecules and ions, and ultimately results in cell lysis and death. EOs are incorporated in food packaging materials in the form of nanoparticles, as the encapsulation supports the stability of EOs in packaging film. The most common EOs are compounds derived from lemongrass, ginger, chamomile, thyme, or the tea tree [173].

## 6. Antibacterial Polymers in Textile Industry and Wearable Electronics

Textiles, made from synthetic polymers, are known for their good resistance to chemicals as well as their durability. On the other hand, they are susceptible to microbial growth on their surface because of retaining moisture, oxygen, warmth, and nutrients from body exudates. Microorganisms such as bacteria, yeast, fungi, and moulds are responsible for diseases in textile users (allergies, infections), and have a harmful effect on textile products, causing staining, odour, and deterioration. In order to maintain hygiene when using synthetic textiles and to prevent the growth of microbes, the textile industry is facing the development of materials that inhibit the growth of bacteria.

One of the proposed solutions is the modification of polyacrylonitrile (PAN), often used in the production of textiles, by immobilizing tetracycline on its surface, which is an effective antibiotic against Gram-positive and Gram-negative bacteria [174]. Another approach to provide antibacterial properties to PAN is the incorporation of copper nanowires and nanoparticles to its surface. The potent antimicrobial properties of copper involve bacterial cell penetration, generation of reactive oxygen species (ROS), metabolite binding, and electrostatic interactions of Cu^2+^ with negatively charged bacteria cells [175].

To protect the artificial textile materials made of polyamide 6 (PA), PET, and PP from bacteria adhesion, the zinc oxide (ZnO) microrods are proposed to be used as an antibacterial layer due to their low toxicity and effectivity as an antibacterial agent. The application of zinc oxide as an antibacterial coating relies on the deposition of ZnO by immersing the fabric in a colloid solution of ZnO nanoparticles [176].

Furthermore, natural compounds such as tannins are gaining attention as antibacterial modifiers in textile industry because of adequate properties such as non-toxicity and good degradation. Tannic acid is an organic acid that belongs to the tannins group and is used as a coating in silk, giving it antibacterial properties. Such coatings are often performed using an adsorption process. Tannic acid exhibits antibacterial activity towards *E. coli* and S. *aureus* [177].

A common natural material used in the textile industry is cotton, also vulnerable to bacteria adhesion. In order to impart antibacterial properties to these kinds of clothing, a coating of polydopamine (PDA) connected with silver nanoparticles (AgNPs) is formed. This treatment gives excellent antibacterial efficiency towards Gram-positive and Gram-negative bacteria [178].

Antibacterial textile materials are of great significance for medical applications, accelerating wound healing and preventing the development of infections. For this purpose, the curcumin-grafted hyaluronic-acid-modified pullulan polymers (Cur-HA-SPu) was elaborated by means of chemical coagulation. Curcumin is known for its antimicrobial and antioxidant properties, but it also enhances tissue formation and remodeling, wound contraction, and collagen deposition. Hyaluronic acid is a natural component of the extracellular matrix, responsible for skin hydration. Thanks to its good biocompatibility, hyaluronic acid is often applied in tissue engineering [179].

Polymers with antibacterial properties are also applied in producing wearable electronic devices. As in many previously described antibacterial approaches, the wearable electronics industry uses silver nanoparticles and nano titanium dioxide. These nanoparticles are successfully applied in the production of nanofiber breathable ionotronic flexible pressure sensors, prepared on the basis of thermoplastic polyurethane (TPU), PET, and polyimide (PI) nanofiber films [180]. One electronic device gaining growing interest is the conductive polymer-based hydrogel (CPHs), which is a soft electronic material applied as wearable and implantable devices, such as a paintable conductive adhesive hydrogel patch. The role of the self-adhesive hydrogel patch is to bind to the surface of a heart, effectively supporting its regenerative abilities. There are also CPHs developed to mimic the mechanical and perception properties of skin. The medical applications of CPHs are possible due to their surface modification with antibacterial coatings, e.g., the nanostructure of polydopamine with silver nanoparticles (PDA@Ag NPs) [181].

The electronic skin produced from hydrogels is modified with tannic acid and quaternary ammonium to give the final product antibacterial properties [182]. For the purposes of quantitative detection and imagining, quantum dots (QDs) with integrated targeting are an excellent solution for drug delivery tracking. After adsorption of crystal violet (CV), the QDs–CV complex is obtained and dispersed in medical-grade polyurethane. The QDs–CV complex is effective in combatting MRSA and *E. coli* [183].

Technological progress forces the development of electrical devices that would operate independently of conventional batteries. Autonomous triboelectric, polypyrrole-based generators with modified cotton are a modern solution in electrically driven antibacterial treatments, showing biocidal effects on *Staphylococcus aureus*. Self-powering nano generators can also be produced with poly(vinylidene fluoride) PVDF as a piezoelectric material, modified with Ag NPs [184].

## 7. Conclusions and Future Prospects

An excessive amount of antibiotics released into the environment has significantly contributed to the development of bacterial resistance to antibiotics [8,9,10,11,12,13]. The use of polymers for antibacterial purposes is becoming more and more popular in many industries where green chemistry and environmental protection are of great importance. There is a growing interest in the use of polymers for antibacterial purposes. Some natural macromolecules, e.g., CTS [17,18,19,20], exhibit intrinsic antibacterial activity, while starch and cellulose require surface modification. In many cases, the antibacterial properties result from the chemical structure of the polymer [16,17,18,19,20], but most synthetic polymers also need to be functionalized to give them antimicrobial properties. In order to meet the requirements of green chemistry, organic compounds of natural origin, i.e., fatty acids [57,58,59] or essential oils [60,61,62], are gaining increasing recognition as modifiers. The mechanisms of surface functionalization according to RAFT [66,67,68,69] and ATRP [70,71,72] polymerisation reactions have been described. AMPs are widely used in medicine due to their synergistic effect with antibiotics [106,107,108,109]. In addition, due to their antifouling activity, they can be successfully used as antibacterial coatings in tissues and prosthetic materials engineering, e.g., PEG, PAM [118], and PEI [134]. In the face of the global water crisis, the research and development of antibacterial membranes for water treatment is crucial for human safety and health. AMPs such as PVDF [139] or polyamide modified with nanoparticles, i.e., DADMAC [140], Ag NPs, and CuO NPs [141,142], find usage for water filter production. In the food industry, polymers such as PVC, PET, PP, and PE [146,147] are used for packaging production. Modified TPU, PET, and PI nanofiber films are applied for the production of wearable antibacterial electronics [171].

Future research perspectives on antibacterial polymers aim at developing positively charged nanoparticles that easily penetrate the bacterial biofilm and are able to precisely release antibiotics in deeper areas of the biofilm, reducing the risk of their uncontrolled distribution into the environment. The antibiotics-loaded superparamagnetic nanoparticles enhance their delivery with the help of a magnetic field [185]. Click chemistry and controlled polymerization techniques have considerably high potential as synthetic routes of antibacterial polymers [186].

## Figures and Tables

**Table 1:** The mechanism of antibacterial action of selected polymers of natural origin.

Natural Polymers with Antibacterial Activity		Mechanism of Antimicrobial Action	Microbes Combated by Polymer	Ref.
**Modified chitosan**	O-modified CTS N-modified CTS N,O-modified CTS	Hydrophobic interactions and chelation, cell wall destruction by protonated amine groups (pH < pK_a_)	*B. subtilis*, *L. monocytogenes*, *B. megaterium*, *B. cereus*, *L. brevis* and *L. bulgaricus*	[21,22]
**Modified cellulose**	Cellulose nanofibers functionalized with AgO nanoparticles	Increase of bacterial cell permeability followed by the leakage of K^+^ and proteins	*S. aureus*, *E. coli*	[23]
	Cellulose fibres functionalised with star-like ZnO	Disruption of the cell membrane due to the reaction with hydrogen ions and ROS formation, triggering the process of bacterial DNA amplification and gene expression	*C. albicans*	[24]
	Cellulose acetate sorbate (CASA)	Inhibiting the microbial enzyme systems due to the presence of hydrophobic sorbic acid, which enables penetration of CASA through the hydrophobic bacteria cell	*E. coli*, *S. aureus*	[25]
**Cationized starch**	Starch modified with halogenated benzene	Establishing a hydrophobic balance, which leads to an enhanced interaction with the surface of the bacterial cell	*E. coli*, *S. aureus*	[26]
	Starch modified with quaternary ammonium	Electrostatic interactions between the bacterial cell and cationic quaternary ammonium, hydrophobic quaternary ammonium chain interaction with Gram-positive bacteria	*E. coli*, *L. monocytogenes*	[27]
	Starch modified with EOs	Triglycerides present in EOs lead to the disintegration of bacterial cell walls, followed by interference with nutrient transport between internal and external mediators	*Gram-positive bacteria*	[28]

## Data Availability

Not applicable.

## Abbreviations

AMP	antimicrobial polymers
ARB	antibiotic resistant bacteria
ARGs	antibiotic resistance genes
ATRP	atom transfer radical polymerization
BA	boswellic acid
CA	cellulose acetate
CPHs	Conductive polymers-based hydrogels
CTS	chitosan
CuAAC	opper (I)-catalyzed azide- alkyne cycloaddition “click” reaction
CV	Cristal violet
DADMAC	diallyldimethyl ammonium chloride
DD	deacetylation degree
DIVEMA	copolymer of maleic anhydride (MA) and divinyl ether (DVE)
DVE	divinyl ether
ECM	extracellular matrix
EM	elastic modulus
EOs	Essential oils
ET	ethyl cellulose
FO	Forward osmosis
FDA	Food and Drug administrator
GO	Graphen oxide
HA	Hydroxyapatite
LDPE	low-density polyethylene
Ly	Lysozyme
MA	maleic anhydride
MC	methyl cellulose
MDRs	multidrug-resistant bacteria
MF	microfiltration
MMA	methyl metacrylate
MMM	mixed matrix membranes
MN	microneedle
MRSA	methicillin resistant *Staphylococcus aureus*
NHS	N-hydroxysuccinimide
NPs	nanoparticles
NF	nanofiltration
PAMAM	poly(amido)amine
PAM	poly (acrylamide)
PAN	polyacrylonitrile
PBC	pentablock copolymer
PCL	poly(epsilon-caprolactone
PDA	polydopamine
PDLA	poly(D-lactide)
PDMS	polydimethylsiloxane
PEI	polyethyleneimine
PET	poly(ethylene tetraphtalate)
PGA	poly(glycolide)
PHMB	polyhexamethylene biguanidine
PHMG	polyhexamethylene guanidine
PI	polyimide
PLGA	poly(lactic-co-glycolic acid)
PP	poypropylene
PPI	polypropylenimine
PSF	polysulfone
PVC	polyvinylchloride
PVP	poly(4- vinylpyridine)
QACs	Quaternary ammonium polymer
QDs	Quantum dots
QGO	Quaternary ammonium salt
RAFT	reversible addition-fragmentation chain transfer
RO	Reversed osmosis
ROS	Reactive oxygen species
SA	Sulfonamides
TA	Tannic acid
TMP	trimethoprim
TPU	Thermoplastic polyurethane
